# Genome-wide identification of membrane-bound fatty acid desaturase genes in *Gossypium hirsutum* and their expressions during abiotic stress

**DOI:** 10.1038/srep45711

**Published:** 2017-04-04

**Authors:** Jiyu Feng, Yating Dong, Wei Liu, Qiuling He, M. K. Daud, Jinhong Chen, Shuijin Zhu

**Affiliations:** 1 Department of Agronomy, Zhejiang University, Hangzhou 310058, China; 2 College of Agronomy, Henan Agricultural University, Zhengzhou 450002, China; 3 Department of Biotechnology and Genetic Engineering, Kohat University of Science and Technology, Kohat 26000, Pakistan

## Abstract

Membrane-bound fatty acid desaturases (FADs) are of great importance and play multiple roles in plant growth and development. In the present study, 39 full-length FAD genes, based on database searches, were identified in tetraploid upland cotton (*Gossypium hirsutum* L.) and were phylogenetically clustered into four subfamilies. Genomic localization revealed that 34 genes were mapped on 22 chromosomes, and five genes were positioned on the scaffold sequences. The FAD genes of *G. hirsutum* in the same subfamily had similar gene structures. The structures of paralogous genes were considerably conserved in exons number and introns length. It was suggested that the FAD gene families in *G. hirsutum* might be duplicated mainly by segmental duplication. Moreover, the FAD genes were differentially expressed in different *G. hirsutum* tissues in response to different levels of salt and cold stresses, as determined by qRT-PCR analysis. The identification and functional analysis of FAD genes in *G. hirsutum* may provide more candidate genes for genetic modification.

Fatty acids, a major source of energy in plants, are the primary constituents of plant membrane phospholipids and store triacylglycerols^1^. In most of the plant tissues, fatty acid biosynthesis occurs in the plastids by a successive concatenation of two carbon long units resulting in the formation of 16- or 18-carbon long fatty acids that predominate in the cellular membranes^2^,^3^. They are over 75% unsaturated and play essential roles like regulating plant growth and responding to variance in environmental signals^4^,^5^,^6^. The unsaturated fatty acids, compared with saturated fatty acid, contain one or more C = C double bonds in their fatty-acyl chains at specific locations^4^.

Membrane-bound Fatty acid desaturases (FADs), encoded by a large gene family responsible for membrane lipid modification and re-ordering^7^, are the key enzymes for inducting one or more carbon double bonds at different positions in the hydrocarbon chains of fatty acids^8^. In the eukaryotic genomes, four large branches of FAD gene family with distinct functions have been identified previously. These are First desaturase, Omega desaturase, Front-end desaturase, and Sphingolipid desaturase^9^. The predominant First desaturase subfamily members are encoded by *Arabidopsis* desaturase (ADS) genes and possess double bonds at Δ7 and Δ9 positions of palmitic acid (16:0) or stearic acid (18:0)^9^,^10^. In Omega desaturase subfamily, a double bond is found between pre-existing double bond and the acyl end at Δ12 and Δ15 desaturases^8^. Typically, the Front-end desaturase subfamily contains a *cis* or *trans* Δ8 double bond produced by sphingoid long-chain bases at Δ8 desaturases (SLDs)^9^,^11^. The sole function of Sphingolipid desaturase subfamily is to modify sphingolipids by causing desaturation at Δ4-position and hydroxylation at the C-4-position. They are also called as dihydroceramide Desaturases (DSDs) due to their highest activity of dihydroceramide^12^.

Nineteen *Gossypium raimondii* FAD genes have been separated into above mentioned four branches and named after corresponding encoding genes^13^. It is known that changing bond number and/or position may form different types of fatty acids^14^,^15^. For instance, introducing of a double bond at Δ12 position of oleic acid (18:1) can produce linoleic acid (18:2)^16^.

To date, several membrane-bound FAD genes from different plant species have been characterized. It is evident by the previous studies that FAD genes are expressed in adverse environmental conditions. For example, in tomato, over-expression of *LeFAD3* gene enhanced the tolerance of tomato seedlings against salt stress^17^, whereas low-expression of *LeFAD7* increased tolerance of tomato leaves to high-temperature (45 °C)^18^. Similarly, over-expression of either *FAD3* or *FAD8* increased tolerance of tobacco plants to drought^19^, and over-expression of *FAD7* alleviated the damage of cold stress in tobacco plants^20^. Moreover, over-expression of omega-3 desaturases improved tolerance of various transgenic plants to both drought and chilling stresses^20^,^21^,^22^,^23^,^24^. In addition, *FAD2* and *FAD6* were induced in seedlings of *Arabidopsis* under salinity stress^25^,^26^. In general, the FAD genes regulate the levels of fatty acid unsaturation in membrane lipids thus improving plant tolerance to adverse environments^8^.

Cotton is an important commercial crop grown worldwide to provide generous supply of natural fiber for textile industry and to provide cottonseeds for food, feed, and bio-fuel productions^4^. It is cultivated mainly in the tropical and subtropical regions. Temperatures below 15 °C can affect the plant growth and development causing yield losses mainly due to poor germination and high seedling mortality. *Gossypium hirsutum* L., an allotetraploid species, is one of the most commonly grown cotton species for commercial production^4^. Originally, it was developed from two ancient diploid cotton species, *G. raimondii* and *G. arboreum*, by hybridization and chromosome doubling about 1–2 MYA^3^. It seems to be an interesting model system not only for the research of genome evolution, but also for studying the roles of polyploid in crop development and domestication with high research value. The fatty acid families from *G. raimondii* and *G. arboreum* have already been identified and characterized^13^. In *G. hirsutum*, Δ12 desaturase (*FAD2*) and Δ15 desaturase (*FAD3* and *FAD7/8*) have been studied, mainly focused on cold adaptation at seedling stages^1^,^2^,^3^,^7^. Furthermore, the *FAD2*-*1* was found to be a seed-specific desaturase responsible for the synthesis of polyunsaturated fatty acids in the cottonseeds^27^, while the *FAD2*-*2* had low expression levels throughout the seed development^2^.

In this study, all the FAD genes in *G. hirsutum* were identified and characterized, and the phylogenetic analysis and structural diversification, as well as the expression profiles of the detected GhFAD genes in response to salt and cold stress regimes across different plant tissues were conducted. This research work may contribute to widen our understanding about the structure and function of FAD gene family in *G. hirsutum*, which may provide some candidate genes for predictable modification of fatty acid profiles in order to improve the plant vigor and seed nutritional value for cotton breeders^1^.

## Results

### Identification of FAD genes in *G. hirsutum*

Based on the completed genome sequences of *G. hirsutum*, a genome-wide search for FAD genes was performed by BlastP and tBlastN program, using *Arabidopsis* and rice FAD genes as the query sequences. Subsequently, all pre-identified proteins were subjected to domain analysis using Pfam and SMART databases for further confirmation, in the presence of FAD protein domain (PF00487). Eventually, a total of 39 FAD genes in *G. hirsutum*, based on their orthologs with reported counterparts in *Arabidopsis*^28^,^29^,^30^, were predicted and annotated (Table 1).

The protein sequences, encoded by these 39 FAD genes in *G. hirsutum*, were detected. They ranged in length from 205 amino acids of *GhFAD2.5A* to 475 amino acids of *GhFAD7A* and *GhFAD2.3D*, with an average of 388 amino acids approximately. Similarly, the predicted molecular weight (Mw) of these deduced proteins varied from 23.76 kDa to 54.27 kDa, and the theoretical isoelectric point (pI) varied from 6.80 to 9.61.

### Phylogenetic relationship among the FAD genes

**Figure 1:**
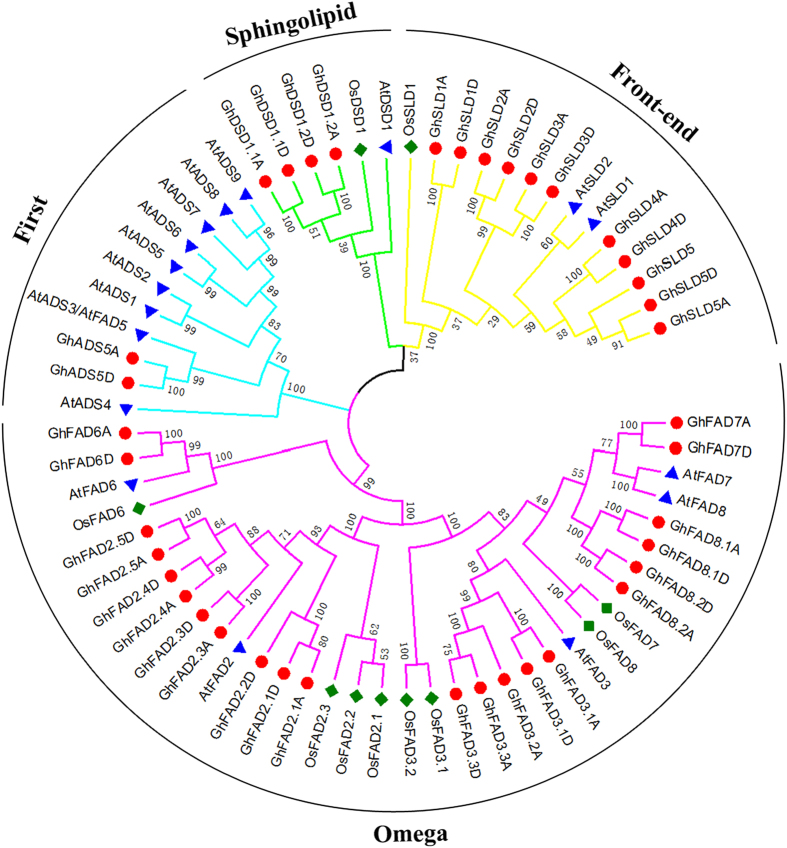
Phylogenetic relationships of FAD genes from *G. hirsutum, Arabidopsis*, and rice. A Neighbor-Joining (NJ) phylogenetic tree of all detected FAD genes was constructed, using MEGA 5.2 program with bootstrap test (replicated 1000 times). The Omega, First, Sphingolipid, and Front-end subfamily were marked in pink, blue, green, and yellow, respectively.

To evaluate the evolutionary relationships among the FAD gene members, all the genes from *G. hirsutum* (39), *Arabidopsis* (17), and rice (10) were aligned separately by Neighboring-Joining method to generate an un-rooted phylogenetic tree (Fig. 1). For confirmation, a phylogenetic tree was also constructed by Maximum Evolution method and the results were found almost consistent with Neighbor-Joining method, showing a little differences at some branches (Supplementary Fig. S1). Further, the FAD genes in these three species were divided into four well-defined monophyletic groups, suggesting the existence of a common ancestor before the divergence of monocots and dicots. The First desaturase group encoded by ADS genes was composed of 11 ADS genes, nine from *Arabidopsis* and two from *G. hirsutum*. No ADS genes were found in the rice, suggesting some evolutionary role in its genome. The ADS genes were either lost in the rice or were acquired by the dicotyledons after divergence from the last common ancestor. In the Omega desaturase group, there were five genes from *Arabidopsis*, eight genes from rice, and 22 genes from *G. hirsutum*. There were 14 SLD genes in the Front-end desaturase group which included 11 gens from *G. hirsutum*, two from *Arabidopsis*, and one from rice. The Sphingolipid desaturase subfamily contained only six DSD genes, four DSD genes from *G. hirsutum*, only one from *Arabidopsis* and rice each. Interestingly, the *G. hirsutum* possessed more gene members than *Arabidopsis* and rice in these three subfamilies except First desaturase in which most of the gene members belonged to *Arabidopsis*.

Though several pairs of paralogous genes in the terminal branches of the *G. hirsutum* phylogenetic tree were distributed in each subfamily, several other FAD genes were not clustered together with the FAD genes from *Arabidopsis* and rice. This suggested that these FAD genes might have merged in *G. hirsutum* genome after diverging from the last common ancestor or have been lost in *Arabidopsis* and rice. Gene numbers in First and Sphingolipid desaturase groups were lower than others, which harbored two and four GhFAD genes, respectively. Taken together, the presence and the absence of species-specific FAD genes were due to the functional divergence. The FAD genes in *G. hirsutum* might have undergone rapid expansion during the course of evolution and exhibited a tendency to cluster into the same subgroup due to the conserved functions.

### Genomic localization of FAD genes

**Figure 2:**
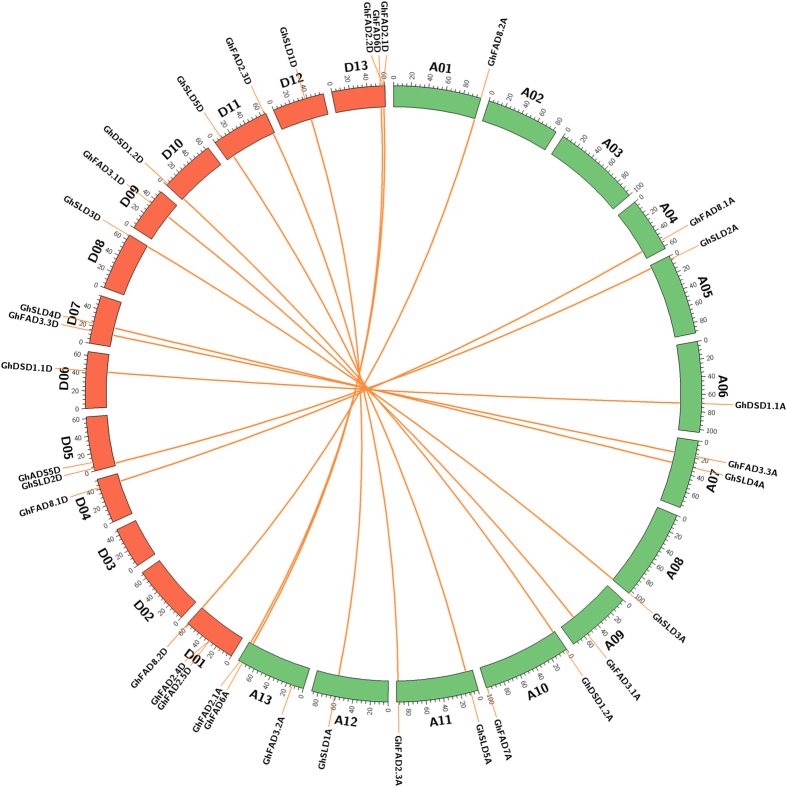
Genomic localization and paralogs of FAD genes of *G. hirsutum*. Chromosomes A and D were indicated with different colors. The paralogous genes belonging to the FAD gene family were connected by orange lines.

Genomic localization of FAD genes in *G. hirsutum* notably revealed that 34 genes were mapped on the 22 chromosomes, except for chromosome A02, A03, D02, and D03 (Fig. 2). The rest five genes, *GhFAD7D, GhFAD2.4A, GhFAD2.5A, GhADS5A*, and *GhSLD5* were positioned on the scaffold sequences. As shown in Fig. 2, chromosome A01, A04, A05, A06, A08, A09, A12, D04, D06, D08, D09, D10, and D12 harbored only a single FAD gene each, while two FAD genes were present on each chromosome of A07, A10, A11, D05, D07, and D11. Moreover, there were three FAD genes on chromosome A13, D01, and D13 each.

### Duplication of FAD genes

To date, the mechanism of FAD genes expansion in *G. hirsutum* remains unclear. We deliberately investigated the relationship between genetic divergence and gene duplication within the FAD gene family of allotetraploid cotton. Total 13 duplicated gene pairs were found in this study, i.e. *GhFAD8.2A/GhFAD8.2D, GhFAD8.1A/GhFAD8.1D, GhSLD2A/GhSLD2D, GhDSD1.1A/GhDSD1.1D, GhFAD3.3A/GhFAD3.3D, GhSLD4A/GhSLD4D, GhSLD3A/GhSLD3D, GhFAD3.1A/GhFAD3.1D, GhDSD1.2A/GhDSD1.2D, GhSLDL5A/GhSLD5D, GhFAD2.3A/GhFAD2.3D, GhSLD1A/GhSLD1D*, and *GhFAD6A/GhFAD6D*. They were positioned on individual chromosome and mainly attributed to the segmental duplication events. Meanwhile, no tandem duplication event was found, which showed adjacently positions with no intervening genes instead. This suggested that the expansion of FAD gene family in *G. hirsutum* was mainly attributed to the segmental duplication events.

### Structural organization of FAD genes

**Figure 3:**
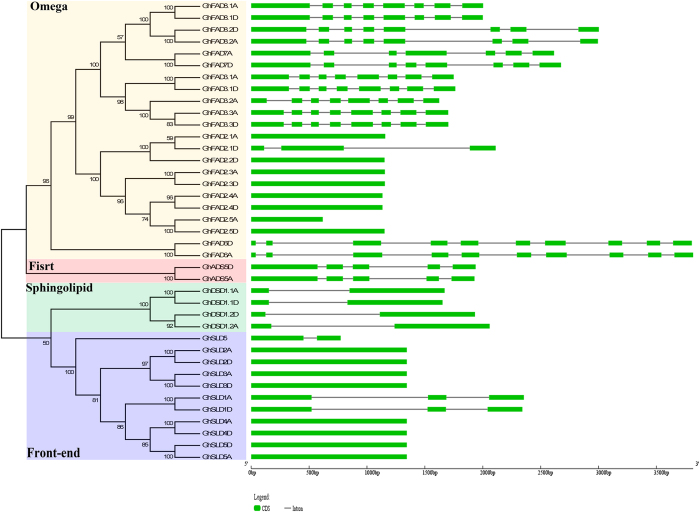
Phylogenetic relationship and gene structure of FAD genes in *G. hirsutum*. Four subfamilies labeled as Omega, First, Sphingolipid, and Front-end were marked with different color backgrounds. Exons were represented by green boxes and introns by grey lines.

In the current experiment, 39 FAD genes were clustered into four subfamilies (Fig. 3). Generally, the FAD genes in the same subfamily had strikingly similar exon/intron structure than other subfamilies. For instance, in the First desaturase subfamily, *GhADS5D* and *GhADS5A* harbored five exons with highly conserved length and four introns in closed size. In contrast, all the genes in the Sphingolipid desaturase subfamily possessed only two same-sized exons. Generally, the genes of the Front-end desaturase subfamily possessed a single exon generally, except *GhSLD5* which had two exons and one shorter intron. However, there were three exons in *GhSLD1A* and *GhSLD1D*. The similar exon-intron structure agreed well with their close phylogenetic relationship. In contrast to these three relatively conserved subfamilies, the FAD genes in the Omega desaturase subfamily showed a complex distribution of exons and introns. For example, the genes of *FAD3, FAD7*, and *FAD8* sections mostly had up to eight exons of relatively conserved length, except for *GhFAD7A* which possessed seven exons. While all the FAD genes in *FAD2* section had a single exon, except for *GhFAD2.1D*, which harbored three exons. In addition, *GhFAD6D* and *GhFAD6A* were composed of ten exons. Among the paralog pairs, uniformity was observed in the gene structures. Since *G.hirsutum* is an allotetraploid plant, the essential integrated evolutionary relationship was done by the sequences of the diploid *G. raimondii* and *G. arboreum* progenitors (Supplementary Fig. S2). The detailed comparison indicated that the exon-intron structures were identical to the result of phylogenetic relationships and were highly conserved in each subfamily, though parts of the orthologous genes demonstrated certain differences.

### Selective pressure analysis of the duplicated FAD genes

To investigate the selective the duplicated FAD gene pairs, the non-synonymous to synonymous substitution ratios (Ka/Ks) were calculated. Generally, Ka/Ks ratio >1 indicates positive selection, Ka/Ks = 1 indicates neutral selection, while a ratio <1 indicates negative or purifying selection. In *G. hirsutum*, the Ka/Ks ratios for 13 duplicated pairs were <1 (Table 2), which suggested that they had experienced strong purifying selection pressure contributing largely to the maintenance of function in *G. hirtusum* FAD gene family.

### Gene expression patterns in different plant tissues

**Figure 4:**
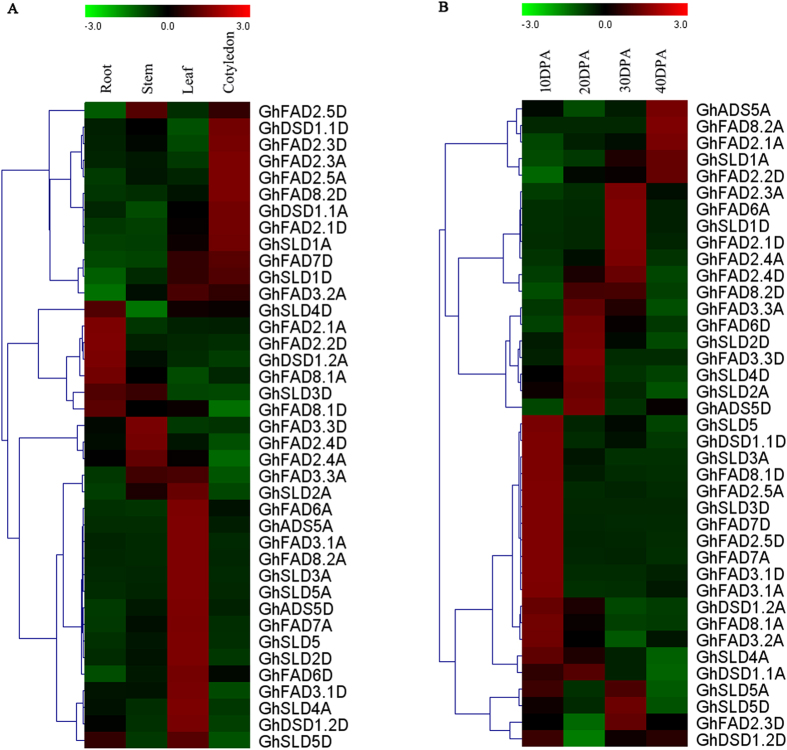
Expression patterns of 39 FAD genes of *G. hirsutum*. (**A**) Expression patterns in four representative tissues. (**B**) Expression patterns in developmental ovules. The color scales represented the relative signal intensity values.

39 FAD genes were multifunctional in different tissues of *G. hirsutum* plant. In order to evaluate their expression patterns, a comprehensive qRT-PCR analysis was performed and distinct tissue-specific expression patterns were observed as illustrated in Fig. 4A. Eight FAD genes, i.e. *GhSLD4D, GhFAD2.1A, GhFAD2.2D, GhDSD1.2A, GhFAD8.1A, GhSLD3D, GhFAD8.1D*, and *GhSLD5D* were preferentially expressed in the roots. In the stems, seven FAD genes, *GhFAD2.5D, GhSLD3D, GhFAD3.3D, GhFAD2.4D, GhFAD2.4A, GhFAD3.3A*, and *GhSLD2A* displayed the highest transcript abundance. In the leaves, 20 FAD genes such as *GhFAD7D, GhFAD3.2A*, and *GhSLD5D* exhibited high expression levels. Four FAD genes, *GhFAD2.1A, GhFAD2.2D, GhDSD1.2A*, and *GhFAD8.1A*, were expressed higher in the roots than other plant tissues, suggesting a potentially specialized function in the roots development or roots activities. While 12 FAD genes, *GhFAD2.5D, GhDSD1.1D, GhFAD2.3D, GhFAD2.3A, GhFAD2.5A, GhFAD8.2D, GhDSD1.1A, GhFAD2.1D, GhSLD1A, GhFAD7D, GhSLD1D*, and *GhFAD3.2A*, exhibited specifically high transcript accumulation in the cotyledons.

There were 18 paralogs among these FAD genes, but only one pairs, *GhFAD2.3A/GhFAD2.3D* clustered together, which inferred that the expression of the FAD paralogs in *G. hirsutum* have experienced divergence.

### Gene expression patterns in developmental ovules

The expressions of the membrane-bound fatty acid genes in different developmental ovules were shown in Fig. 4B. Eleven FAD genes, *GhSLD5, GhDSD1.1D, GhSLD3A, GhFAD8.1D, GhFAD2.5A, GhSLD3D, GhFAD7D, GhFAD2.5D, GhFAD7A, GhFAD3.1D*, and *GhFAD3.1A* shared the characteristic of having maximal expressions in early stage (10day post anthesis, DPA) and lower expressions at later developmental stages. In addition, *GhFAD2.4D, GhFAD8.2D*, and *GhFAD3.3A* exhibited specifically high transcript accumulation in ovule development later, which was focused on 20 and 30 DPA. *GhADS5A, GhFAD8.2A, GhFAD2.1A, GhSLD1A*, and *GhFAD2.2D* had similar expression behavior of higher expression on 40 DPA stages. However, *GhFAD6D, GhSLD2D, GhFAD3.3D, GhSLD4D, GhSLD2A, and GhADS5D* were strongly expressed on 20 DPA. Notably, the subfamily of *GhFAD2*, comprised of *GhFAD2.3A, GhFAD2.1D, GhFAD2.4A*, and *GhFAD2.4D* showed strikingly consistent expression patterns in later developmental stages (30 DPA), which clued to the existence of functional conservation.

### Gene expression patterns under salt and cold stresses

**Figure 5:**
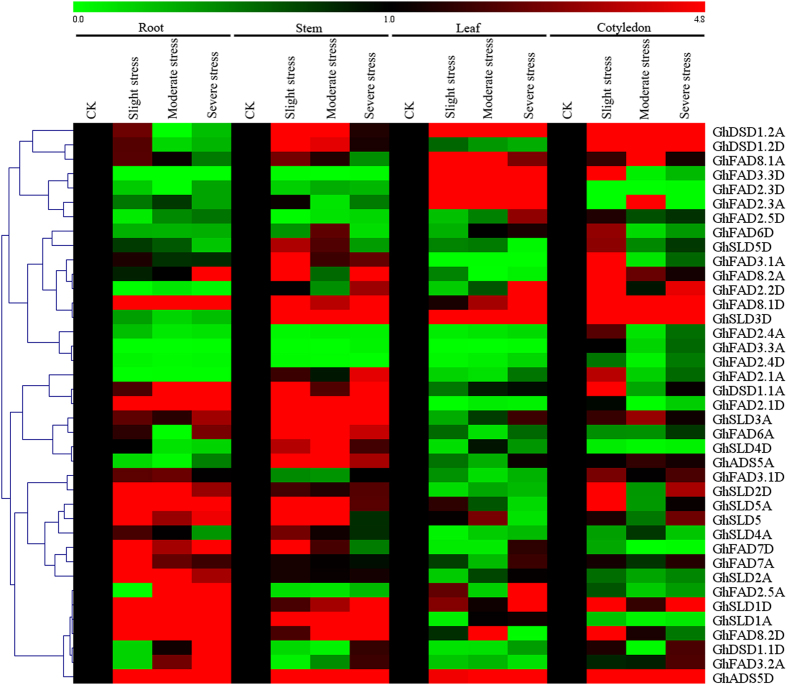
Expression patterns of 39 FAD genes of *G. hirsutum* under gradient salt stress. The color scales represented the relative signal intensity values.

Gene expressions for all the FAD genes were also observed in different tissues under different levels of salt and cold stresses. Results showed that the salt stress caused changes in the expression patterns in the form of induction or suppression (Fig. 5). In the roots, twelve FAD genes, *GhFAD8.1D, GhDSD1.1A, GhFAD2.1D, GhSLD2D, GhSLD5A, GhSLD5, GhFAD7D, GhSLD2A, GhSLD1D, GhSLD1A, GhFAD8.2D*, and *GhADS5D*, were up-regulated under different levels of salt stress. In the stems, most of the FAD genes were highly expressed after salt treatment, while *GhFAD3.3D, GhFAD2.3D, GhFAD2.5D, GhFAD2.4A, GhFAD3.3A, GhFAD2.4D*, and *GhFAD2.5A* were less expressed over all stages. However, compared with the stems, only few FAD genes were up-regulated in the leaves, but higher expressions of *GhFAD3.3D, GhFAD3.1A, GhFAD2.1A, GhDSD1.1A*, and *GhSLD5A* were found under slight salt stress in the cotyledons. Among all, only *GhADS5D* was highly expressed in all tissues under salt stress. The expressions of *GhFAD8.2A* in the roots and *GhFAD2.2D* in the leaves were relatively low at slight stress but showed dramatic increases during severe stress. However, *GhDSD1.2A, GhDSD1.2D, GhSLD5A, GhSLD5, GhSLD4A*, and *GhFAD7D* in the stems showed the highest transcript abundance after stress then decreased thereafter. Furthermore, four paralogs, *GhDSD1.2A/GhDSD1.2D, GhFAD2.3D/GhFAD2.3A, GhFAD7A/GhFAD7D*, and *GhSLD1A/GhSLD1D*, were clustered together having similar expression patterns.

**Figure 6:**
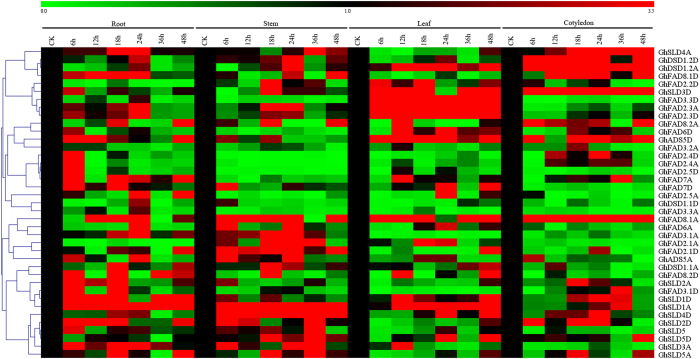
Expression patterns of 39 FAD genes of *G. hirsutum* under gradient cold stress. The color scales represents the relative signal intensity values.

The gene expressions of membrane-bound fatty acids in different plant tissues under different low temperature levels were shown in Fig. 6. In all the tested tissues, the expression level of *GhFAD8.1A* remained very high under low temperature as compared with other genes, suggesting its potential roles in tissue development and/or function. In the roots, *GhFAD2.2D, GhFAD3.2A*, and *GhFAD2.1A* transcripts were largely restricted, whereas the transcripts of *GhDSD1.2D, GhDSD1.2A, GhFAD2.5A, GhDSD1.1D*, and *GhFAD3.3A* were increased to their peaks at 24 hours interval after cold treatment. In contrast, *GhSLD1A* and *GhSLD4D* showed the highest transcript abundance, whereas *GhFAD3.3D, GhFAD6D, GhFAD3.2A, GhFAD2.4D, GhFAD2.4A, GhFAD2.5D, GhFAD7A, GhFAD2.5A*, and *GhFAD3.3A* exhibited low expressions in the stems. In addition, the membrane-bound fatty acid genes were extensively expressed in the leaves and cotyledons, especially for those of *GhFAD3.3D, GhFAD2.3A*, and *GhFAD2.3D* in the leaves and *GhDSD1.2A, GhSLD3D*, and *GhFAD8.1A* in the cotyledons, which were significantly up-regulated when the salt stress was applied for a longer time. Several fatty acid genes in the cotyledons, such as *GhFAD3.3D, GhFAD2.5D, GhFAD7D*, and *GhFAD3.3A*, were suppressed by cold stress application.

## Discussion

*G. hirsutum* is a natural allotetraploid produced by the interspecific hybridization of A- and D-genome diploid progenitor species. Based on the genome scans of several plant genomes, the FAD gene family has been systematically investigated in *Arabidopsis* (17), soybean (10)^31^, *G. raimondii* (19), and *G. arboreum* (20)^13^. In current work, a total of 39 putative FAD genes were identified in the genomes of *G. hirsutum*, which contained more FAD genes than other plant species such as *Arabidopsis* and rice. It might be mainly due to the recent polyploidy and segmental duplication events in *G. hirsutum* evolutionary history. Coincidentally, the number of FAD genes in *G. hirsutum* (AD_1_ allotetraploid) was exactly the sum of those in *G. raimondii* (19) and *G. arboretum* (20). Moreover, all these FADs were distinctly classified into four groups as Omega desaturase, First desaturase, Sphingolipid desaturase, and Front-end desaturase consistently. In three subfamilies, Front-end desaturase, Omega desaturase, and Sphingolipid desaturase, *G. hirsutum* had much more FAD genes than *Arabidopsis* and rice, which implied that these three subfamilies arose before the divergence of monocots-dicots and might have undergone species specific expansion process. Furthermore, the sizes of the GhFAD proteins varied markedly, as well as the predicted isoelectric points, suggesting that different GhFAD proteins might have functions in different microenvironments.

Gene duplication plays an irreplaceable role in the process of gene family expansion in the genomes. In *G. hirsutum*, 13 segmental duplicated gene pairs were found, such as five gene pairs in the Front-end desaturase subfamily, two gene pairs in the Sphingolipid desaturase subfamily, and six gene pairs in the Omega desaturase subfamily. These results suggested that the expansion of Sphingolipid desaturase, Front-end desaturase, and Omega desaturase subfamilies were due to the segmental duplication, which was consistent with the previous report^13^, namely the increasing size of FAD genes might be contributed by the segmental duplication events.

It was noteworthy that paralogous FAD gene pairs demonstrated very similar exon/intron distribution patterns in terms of exon number and intron length. For instance, the FAD genes in the First desaturase subfamily and the Sphingolipid desaturase subfamily of three cotton species were highly conserved and clustered distinctly. However, their expression patterns were greatly divergent. It might have involved in the mechanism of adaptation to different environmental conditions. In the current study, different expression patterns of FAD genes were observed in each tissue. G*hADS5A, GhFAD3.1A*, and *GhSLD3A* were expressed markedly at high levels in the leaves but lower in the other tissues, while *GhFAD6D* was constitutively expressed at high levels in all the tested tissues. The tissue specific FAD gene expression patterns observed in the present study indicated their functional divergence during plant development and growth, which needs further study to identify their actual functions. In addition, most of the *FAD2* subfamily such as *GhFAD2.3A, GhFAD2.1D, GhFAD2.4A, GhFAD2.4D, GhFAD2.1A*, and *GhFAD2.2D* showed dramatically high abundance in the process of lipid accumulation. It suggested that these genes were seed-specific responsible for the polyunsaturated fatty acids in the seed oil of cultivated cotton. In addition, the genes which were highly expressed on 20 DPA recruited all types of FAD genes for the biogenesis in different positions or lengths. However, the other development stages involved only three subfamilies, which suggested that 20 DPA might be an important stage for ovule development.

Salinity, resulting mainly due to NaCl, is one of the common environmental stresses that afflicts the growth and yield of crops in many regions all over the world^6^,^26^,^32^,^33^,^34^. In previous studies, *FAD2* and *FAD6* were revealed to be required for salt tolerance in *Arabidopsis*^26^,^32^, while *LeFAD3* up-regulation could alleviate salt stress in early seedling stage^23^. In addition, antisense *AtFAD7* expression resulted in lower salt tolerance in transgenic tobacco plants^6^. In this study, low expression level of *GhFAD2.4D* was found in all tissues treated by salt stress. *GhFAD8.1D* and *GhADS5D* genes were significantly up-regulated in the leaves in response to salt stress, which suggested that these FAD genes might adjust to appropriate levels to protect the cell membrane of cotton plant from salt stress. Low temperature is another serious stress, which significantly affects plant growth and development. The *FAD2*-*3* and *FAD2*-*4* genes from *G. hirsutum* were found to participate in the membrane adaptation to cold stress and induced significantly higher in a previous study^7^. Similarly, the over-expression of *FAD8* could also alleviate the damage of cold stress^35^. In this study, *GhFAD8.1A* was highly expressed implying its potential role in the plant tolerance to low temperature stress, which is consistent with the previous findings^3^,^35^,^36^.

## Conclusion

In the current work, 39 full-length FAD genes, classified into four well-documented groups in conserved exon/intron structures, have been identified and functionally validated in *G. hirsutum*. Most *GhFAD2* subfamily members showed high abundance in later developmental stages, it might have contributed to the accumulation of oil contents in seeds and can be used as target genes to improve cottonseed quality. qRT-PCR analysis also revealed the corresponding expressions of GhFAD genes under different levels of salt and cold stresses. *GhFAD8.1D* and *GhADS5D* exhibited specifically high transcript accumulation in the tested tissues exposed to different levels of cold stress, which suggested that they might be required for salt tolerance. Meanwhile, *GhFAD8.1A* was likely to enhance the plant tolerance to cold stress according to the significant up-regulation after long periods of treatment. The comprehensive investigation carried out in this study may improve our understanding about the involvement of FAD genes to tolerate adverse environments, and can also provide the candidate genes for functional studies.

## Materials and Methods

### Sequence retrieval and genome-wide identification

The genome of *G. hirsutum* was downloaded from MASCOTTON database (http://mascotton.njau.edu.cn/), while the published FAD proteins of *Arabidopsis* and *Oryza sativa* (Supplementary Table S1)^31^,^37^ were retrieved from The *Arabidopsis* Information Resource (TAIR release 10, http://www.arabidopsis.org) and the Rice Genome Annotation Project Database (RGAP release 10, http://riceplantbiology.msu.edu/index.shtml), respectively. BlastP and tBlastN programs were applied to search for the initial identification of the candidate FAD genes of *G. hirsutum* in default parameters, with the queries of *Arabidopsis* and rice FAD protein sequences. Then, the Pfam (http://pfam.sanger.ac.uk/search)^38^ and SMART databases (http://smart.embl-heidelberg.de/)^39^ were used to confirm every putative FADs of *G. hirsutum* with the domain of Pfam FAD gene family (PF00487). Furthermore, the theoretical molecular weight (Mw) and isoelectric point (pI) of the full-length proteins were predicted by using pI/Mw tool (http://web.expasy.org/protparam/)^40^.

### Phylogenetic construction

Clustal X version 2.0^41^ was used to carry out the multiple sequence alignment of full length protein sequences from three species, *G. hirsutum, Arabidopsis*, and rice. As soon as a MAGA file was generated, MEGA 5.2^42^ was further applied to construct an unrooted Neighbor-Joining phylogenetic tree with pairwise deletion option and Possion correction model. Bootstrap test was carried out with 1000 replicates for the reliability of interior branches. To confirm the consistency with the Neighbor-Joining method, the phylogenetic tree was also performed with Maximum Evolution method.

### Chromosomal locations, gene duplications, and structural analysis

The genes were mapped on *G. hirsutum* chromosomes using the Circos tool^43^. Gene duplication of FAD genes in *G. hirsutum* was indicated by (1) shared aligned sequence covering >80% of the longer gene; (2) similarity of the aligned regions >80%; and (3) only one duplicated event was counted for tightly linked genes^44^,^45^. Referring to the different chromosomal locations (Supplementary Table S2), these FAD genes were designated as either tandem duplications or segmental duplications.

Comparing the predicted coding sequences (CDSs) with their corresponding genomic sequences, the exon/intron organization of the individual GhFAD genes were illustrated using the Gene Structure Display Server (GSDS, http://gsds1.cbi.pku.edu.cn/).

### Estimating Ka/Ks ratios for duplicated gene pairs

Initially, all the full-length gene sequences of the duplicated FAD gene pairs of *G. hirsutum* were aligned by Clustal X 2.0 program^41^ and then the non-synonymous (Ka) and synonymous substitution rates (Ks) were calculated using DnaSp V5.0^46^ software. Finally, the selection pressure of each gene pair was assessed based on the Ka/Ks ratio.

### Plant materials and abiotic stress treatment

TM-1, a genetic standard line of *G. hirsutum* was grown in a temperature-controlled chamber adjusted to 28 °C temperature, 70% relative humidity, and 16/8 hours photoperiod. After five days, half of the germinated cottonseeds were transplanted to black plastic buckets containing MS medium (5 liters). After adaption for ten days in the hydroponics, the MS solutions were adjusted to different salt treatments as 0 (control), 0.3% (slight stress), 0.6% (moderate stress), and 0.9% (severe stress). Three biological replicates were carried out for each treatment. After two weeks of salt treatment, the roots, stems, cotyledons, and leaves were sampled, frozen immediately in the liquid nitrogen, and stored at −80 °C for further analyses.

For cold stress, 30 days old seedlings were transferred to a temperature-controlled chamber adjusted to 10 °C. The plants were harvested at 0, 6, 12, 18, 24, 36, and 48 hours cold intervals to conduct expression analysis. Three biological replicates of each sample were also implemented. All collected samples were immediately frozen in the liquid nitrogen and then stored at −80 °C.

In addition, the flowers of TM-1, planted in the fields in Agricultural station of Zhejiang University, Hangzhou, in 2015, were labeled at 0 DPA. The cotton embryos were harvested at 10, 20, 30, and 40 DPA for analysis of gene expression patterns during the seed development. All collected samples were immediately frozen in the liquid nitrogen and stored at −80 °C.

### RNA isolation and qRT-PCR

Total RNA of all collected samples was extracted using the EASYspin Plus Total RNA Extraction Kit (Aidlab, Beijing, China) according to the manufacturer protocol. The NanoDrop 2000 Spectrophotometer was used to determine the quantity and quality of RNAs. Subsequently, using the PrimeScript^TM^ 1st Strand cDNA Synthesis Kit (TakaRa, Dalian, China) to synthesize first-strand cDNAs. The specific primers were designed according to CDSs (Supplementary Table S3). All melting curves produced by qRT-PCR were shown in Supplementary Fig. S3.

The amplification reactions of qRT-PCR were performed with Lightcycler 96 system (Roche) using SYBR the premix Ex taq (TakaRa) in 20 μL volume with following parameters: initializing denaturation at 95 °C for 30 seconds, following 45 cycles denaturation at 95 °C for 10 seconds; annealing at 53–54 °C for 10 seconds, and extension at 72 °C for 20 seconds. In addition, the default setting for the melting curve stag was chosen. Three biological replicates were maintained for each collected sample. The relative expression levels were calculated according to the 2^−ΔΔCt^ method^47^. The heatmap for expression profiles were generated with the Mev 4.0 software^48^ with Pearson correction and complete linkage clustering.

## Additional Information

**How to cite this article**: Feng, J. *et al*. Genome-wide identification of membrane-bound fatty acid desaturase genes in *Gossypium hirsutum* and their expressions during abiotic stress. *Sci. Rep.* **7**, 45711; doi: 10.1038/srep45711 (2017).

**Publisher's note:** Springer Nature remains neutral with regard to jurisdictional claims in published maps and institutional affiliations.

## Supplementary Material

Supplementary Information

## Figures and Tables

**Table 1:** Basic information regarding the FAD genes in *G. hirsutum*.

No.	Gene name	Locus ID	Genomics position	Protein length	Mw(KDa)	PI	Strand
1	GhFAD7A	Gh_A10G2136	A10: 100162007-100164622	475	54.27	9.10	minus
2	GhFAD7D	Gh_D10G2457	scaffold4395_D10: 144858-147533	450	51.15	8.91	minus
3	GhFAD8.1A	Gh_A04G0783	A04: 52670441-52672443	446	50.96	8.47	minus
4	GhFAD8.1D	Gh_D04G1274	D04: 41938588-41940588	446	50.72	8.52	minus
5	GhFAD8.2D	Gh_D01G2221	D01: 61104419-61107421	435	50.40	7.42	plus
6	GhFAD8.2A	Gh_A01G1961	A01: 99569646-99572640	427	49.53	8.17	plus
7	GhFAD3.1A	Gh_A09G0848	A09: 55929662-55931410	388	45.03	9.05	plus
8	GhFAD3.1D	Gh_D09G0870	D09: 33787984-33789746	388	45.11	9.05	plus
9	GhFAD3.2A	Gh_A13G0564	A13: 13306656-13308279	308	35.41	8.69	plus
10	GhFAD3.3A	Gh_A07G0946	A07: 17618250-17619951	376	43.52	8.95	minus
11	GhFAD3.3D	Gh_D07G1026	D07: 14269363-14271065	376	43.55	8.69	minus
12	GhFAD2.1A	Gh_A13G1850	A13: 78167608-78168765	385	44.06	9.09	minus
13	GhFAD2.1D	Gh_D13G2237	D13: 58467204-58469315	291	33.52	9.61	minus
14	GhFAD2.2D	Gh_D13G2238	D13: 58471954-58473105	383	43.89	8.95	minus
15	GhFAD2.3A	Gh_A11G2814	A11: 91511504-91512658	384	44.25	8.96	plus
16	GhFAD2.3D	Gh_D11G3169	D11: 64332280-64333434	475	54.27	9.10	plus
17	GhFAD2.4A	Gh_A01G2094	scaffold112_A01: 53713-54846	377	43.84	8.98	plus
18	GhFAD2.4D	Gh_D01G1227	D01: 30322983-30324116	377	43.66	8.95	minus
19	GhFAD2.5A	Gh_A01G2091	scaffold111_A01: 182694-185218	205	23.76	9.48	plus
20	GhFAD2.5D	Gh_D01G1226	D01: 30279978-30281129	383	44.27	8.94	minus
21	GhFAD6D	Gh_D13G1979	D13: 55126909-55130714	442	51.32	9.17	minus
22	GhFAD6A	Gh_A13G1619	A13: 75012308-75016124	445	51.59	9.09	minus
23	GhADS5D	Gh_D05G1179	D05: 10139919-10141857	386	44.24	9.37	plus
24	GhADS5A	Gh_A05G3863	scaffold1234_A05: 29739-31667	386	44.20	9.37	minus
25	GhDSD1.1A	Gh_A06G1118	A06: 70479575-70481245	324	37.74	6.97	plus
26	GhDSD1.1D	Gh_D06G1371	D06: 42795712-42797364	324	37.72	6.95	plus
27	GhDSD1.2D	Gh_D10G0219	D10: 1935044-1936976	314	36.79	8.68	plus
28	GhDSD1.2A	Gh_A10G0240	A10: 2107036-2109095	331	38.62	8.82	plus
29	GhSLD1A	Gh_A12G0984	A12: 61000719-61003073	326	37.18	6.80	plus
30	GhSLD1D	Gh_D12G1104	D12: 37533014-37535355	326	37.10	7.27	plus
31	GhSLD2A	Gh_A05G0325	A05: 3576223-3577566	447	51.15	8.68	plus
32	GhSLD2D	Gh_D05G0430	D05: 3482279-3483622	447	51.20	8.68	plus
33	GhSLD3A	Gh_A08G2217	A08: 103131115-103132458	447	51.20	8.64	plus
34	GhSLD3D	Gh_D08G2583	D08: 65460731-65462074	447	51.27	8.61	plus
35	GhSLD4A	Gh_A07G1291	A07: 30625403-30626746	447	51.18	8.36	plus
36	GhSLD4D	Gh_D07G1405	D07: 23118528-23119871	447	51.18	8.55	plus
37	GhSLD5	Gh_Sca050655G01	scaffold50655: 42-814	218	25.04	9.03	plus
38	GhSLD5D	Gh_D11G0983	D11: 8582726-8584069	447	51.37	8.94	plus
39	GhSLD5A	Gh_A11G0840	A11: 8503404-8504747	388	45.03	9.05	plus

**Table 2:** Ka/Ks analysis for the duplicated FAD gene pairs of *G. hirsutum*.

Duplicated gene 1	Duplicated gene 2	ka	Ks	ka/ks	Purifying selection	Duplicated type
*GhFAD8.1A*	*GhFAD8.1D*	0.011	0.056	0.199	Yes	Segmental
*GhFAD8.2D*	*GhFAD8.2A*	0.014	0.021	0.670	Yes	Segmental
*GhFAD3.1A*	*GhFAD3.1D*	0.002	0.016	0.139	Yes	Segmental
*GhFAD3.3A*	*GhFAD3.3D*	0.010	0.032	0.320	Yes	Segmental
*GhFAD2.3A*	*GhFAD2.3D*	0.006	0.045	0.128	Yes	Segmental
*GhFAD6D*	*GhFAD6A*	0.003	0.028	0.104	Yes	Segmental
*GhSLD2A*	*GhSLD2D*	0.006	0.028	0.206	Yes	Segmental
*GhSLD3A*	*GhSLD3D*	0.007	0.039	0.174	Yes	Segmental
*GhSLD1A*	*GhSLD1D*	0.013	0.034	0.382	Yes	Segmental
*GhSLD4A*	*GhSLD4D*	0.001	0.024	0.043	Yes	Segmental
*GhSLD5D*	*GhSLD5A*	0.005	0.014	0.356	Yes	Segmental
*GhDSD1.1A*	*GhDSD1.1D*	0.007	0.033	0.212	Yes	Segmental
*GhDSD1.2A*	*GhDSD1.2D*	0.004	0.054	0.074	Yes	Segmental
