# Supplementary material for: Genome-wide identification of membrane-bound fatty acid desaturase genes in Gossypium hirsutum and their expressions during abiotic stress
Source: Sci Rep. 2017 Apr 4;7:45711. doi: 10.1038/srep45711 (PMC5379561; doi:10.1038/srep45711)
Supplement: Supplementary Information [file srep45711-s1.docx]

**Supplementary Information:**

**Genome-wide identification of membrane-bound fatty acid desaturase genes in *Gossypium hirsutum* and their expressions during abiotic stress**

Jiyu Feng^1^, Yating Dong^1^, Wei Liu^2^, Qiuling He^1^, M.K. Daud ^3^, Jinhong Chen^1^, Shuijin Zhu^1,^ *

^1^ Department of Agronomy, Zhejiang University, Hangzhou 310058, China

^2^ College of Agronomy, Henan Agricultural University, Zhengzhou 450002, China

^3^ Department of Biotechnology and Genetic Engineering, Kohat University of Science and Technology, Kohat 26000, Pakistan

*Correspondence and requests for materials should be addressed to S.Z. (email: shjzhu@zju.edu.cn)

**Supplementary Table S1. The FAD genes in Arabidopsis and rice.**

| **Species** | **Gene locus** | **Gene symbol** |
| --- | --- | --- |
| *Arabidopsis thaliana* | AT3G12120 | *AtFAD2* |
|  | AT4G30950 | *AtFAD6* |
|  | AT2G29980 | *AtFAD3* |
|  | AT3G11170 | *AtFAD7* |
|  | AT5G05580 | *AtFAD8* |
|  | AT4G04930 | *AtDSD1* |
|  | AT3G61580 | *AtSLD1* |
|  | AT2G46210 | *AtSLD2* |
|  | AT1G06080 | *AtADS1* |
|  | AT2G31360 | *AtADS2* |
|  | AT3G15850 | *AtADS3/AtFAD5* |
|  | AT3G15870 | *AtADS4* |
|  | AT1G06350 | *AtADS5* |
|  | AT1G06360 | *AtADS6* |
|  | AT1G06100 | *AtADS7* |
|  | AT1G06090 | *AtADS8* |
|  | AT1G06120 | *AtADS9* |
| *Oryza sativa* | LOC_Os02g48560 | *OsFAD2.1* |
|  | LOC_Os07g23410 | *OsFAD2.2* |
|  | LOC_Os07g23430 | *OsFAD2.3* |
|  | LOC_Os08g34220 | *OsFAD6* |
|  | LOC_Os12g01370 | *OsFAD3.1* |
|  | LOC_Os11g01340 | *OsFAD3.2* |
|  | LOC_Os03g18070 | *OsFAD7* |
|  | LOC_Os07g49310 | *OsFAD8* |
|  | LOC_Os02g42660 | *OsDSD1* |
|  | LOC_Os09g16920 | *OsSLD1* |

**Supplementary Table S2. The references of FADs localizations of *G. hirsutum*.**

| **No.** | **Gene name** | **starting point** | **Chromosome localization** | **Chromosome length(bp)** | **Chromosome color** |
| --- | --- | --- | --- | --- | --- |
| 1 | GhFAD7A | 100162007 | A10 | 100866604 | green |
| 2 | GhFAD7D | 144858 | scaffold |  |  |
| 3 | GhFAD8.1A | 52670441 | A04 | 62913772 | green |
| 4 | GhFAD8.1D | 41938588 | D04 | 51454130 | orange |
| 5 | GhFAD8.2D | 61104419 | D01 | 61456009 | orange |
| 6 | GhFAD8.2A | 99569646 | A01 | 99884700 | green |
| 7 | GhFAD3.1A | 55929662 | A09 | 74999931 | green |
| 8 | GhFAD3.1D | 33787984 | D09 | 50995436 | orange |
| 9 | GhFAD3.2A | 13306656 | A13 | 79961121 | green |
| 10 | GhFAD3.3A | 17618250 | A07 | 78251018 | green |
| 11 | GhFAD3.3D | 14269363 | D07 | 55312611 | orange |
| 12 | GhFAD2.1A | 78167608 | A13 | 79961121 | green |
| 13 | GhFAD2.1D | 58467204 | D13 | 60534298 | orange |
| 14 | GhFAD2.2D | 58471954 | D13 | 60534298 | orange |
| 15 | GhFAD2.3A | 91511504 | A11 | 93316192 | green |
| 16 | GhFAD2.3D | 64332280 | D11 | 66087774 | orange |
| 17 | GhFAD2.4A | 53713 | scaffold |  |  |
| 18 | GhFAD2.4D | 30322983 | D01 | 61456009 | orange |
| 19 | GhFAD2.5A | 182694 | scaffold |  |  |
| 20 | GhFAD2.5D | 30279978 | D01 | 61456009 | orange |
| 21 | GhFAD6D | 55126909 | D13 | 60534298 | orange |
| 22 | GhFAD6A | 75012308 | A13 | 79961121 | green |
| 23 | GhADS5D | 10139919 | D05 | 61933047 | orange |
| 24 | GhADS5A | 29739 | scaffold |  |  |
| 25 | GhDSD1.1A | 70479575 | A06 | 103170444 | green |
| 26 | GhDSD1.1D | 42795712 | D06 | 64294643 | orange |
| 27 | GhDSD1.2D | 1935044 | D10 | 63374666 | orange |
| 28 | GhDSD1.2A | 2107036 | A10 | 100866604 | green |
| 29 | GhSLD1A | 61000719 | A12 | 87484866 | green |
| 30 | GhSLD1D | 37533014 | D12 | 59109837 | orange |
| 31 | GhSLD2A | 3576223 | A05 | 92047023 | green |
| 32 | GhSLD2D | 3482279 | D05 | 61933047 | orange |
| 33 | GhSLD3A | 103131115 | A08 | 103626341 | green |
| 34 | GhSLD3D | 65460731 | D08 | 65894135 | orange |
| 35 | GhSLD4A | 30625403 | A07 | 78251018 | green |
| 36 | GhSLD4D | 23118528 | D07 | 55312611 | orange |
| 37 | GhSLD5 | 42 | scaffold |  |  |
| 38 | GhSLD5D | 8582726 | D11 | 66087774 | orange |
| 39 | GhSLD5A | 8503404 | A11 | 93316192 | green |

**Supplementary Table S3. PCR primers and their product sizes in this study.**

| **Gene name** | **Forward primer (5'-3')** | **Reverse primer (5'-3')** | **Size (bp)** |
| --- | --- | --- | --- |
| *GhFAD7A* | TAAAGGTAGAAACTTAGGGGGG | GGATCTTTAACCCAACAATG | 204 |
| *GhFAD7D* | TGCCGTCAAAATCCACCAT | CATTATTAACCCCATCAGTGGT | 230 |
| *GhFAD8.1A* | TTGTCCCGACCGAACGAA | CCATCCAGCCACATCACG | 154 |
| *GhFAD8.1D* | CTTGTATCTACGATAGACCCGC | TAAATGGGGCACTCACGT | 150 |
| *GhFAD8.2D* | GGGGCATCTATTGCATTCTTT | GGGGAAAGGAAACTTGAACC | 181 |
| *GhFAD8.2A* | CCTCTTCCTCGTATCTGC | CAACTTTCAATGGCGTAG | 127 |
| *GhFAD3.1A* | AATAGTTTGAGCAGCAGGAC | TGGACCGATTACAGAGGATA | 225 |
| *GhFAD3.1D* | GCACAGAAGTCCAGGAAAG | TCATGGATGGACCGATTACAA | 155 |
| *GhFAD3.2A* | GAAAAGGATGAGTCATGGGTG | CATTGGAGACCAACAGGCAT | 228 |
| *GhFAD3.3A* | ATGGGTTCCGTTGCCTGAG | CTTCTTTGCCTGGGCTTCT | 128 |
| *GhFAD3.3D* | TTGGGCTGTCTTTGTCCT | CGTGAATCGCAGTAATCG | 244 |
| *GhFAD2.1A* | GGGGTGCCTCTACTTATTGT | ATGCGTATCGGTGATGTTAT | 180 |
| *GhFAD2.1D* | GTACTTAGCCTTCAACGTTTCA | CCAGCCCTTTTGTTGCAGT | 164 |
| *GhFAD2.2D* | TTATTGTGAATGCCTTCCTTG | ATCGGTTTGATTGCTTTAGTG | 238 |
| *GhFAD2.3A* | GAAACAAGAATCGGGCTCA | TGTGGAAGTAAGTGGTGGC | 185 |
| *GhFAD2.3D* | GCAGGTGGCAGAATGTCG | GAGGGAGGTTGCGGAAGT | 229 |
| *GhFAD2.4A* | ACCTTCCTCAGCCTCTCTCCT | TTCGAGGGAACCGGTATTG | 230 |
| *GhFAD2.4D* | ACTTCCCTAACCTTCCTCAGG | TCGAGGGAACCGGTGTTA | 238 |
| *GhFAD2.5A* | TACTTCCCTAGCCTTCCTCAAC | GTGTTGGAATGGTGACGG | 227 |
| *GhFAD2.5D* | TTCCAACACTGGTTCCCTC | AGGGTCATAGTGGCAAGCTAAC | 202 |
| *GhFAD6D* | CTTTGTTATAGGCCACGATTGT | GTGCCAAGCTGTATCTTCTGATAA | 175 |
| *GhFAD6A* | GTGGGACAATCTTCTCGT | ATCCATAAGTTTCCGCTA | 161 |
| *GhADS5D* | AAAGCAGAGTCCTTTGGGC | AAATTCTACCCTGGGTTTTC | 140 |
| *GhADS5A* | AGCATTGTTAAAGCCCAACT | TTAGCCAAAGCCGACACT | 213 |
| *GhDSD1.1A* | ATCAACTTAGCTATTCAGGTCGC | CTCGGAGATGAAATGACCG | 135 |
| *GhDSD1.1D* | ACCGAAGCCCAATTCGTA | GCTCGGAGATGAAATGACCT | 256 |
| *GhDSD1.2D* | TTTTCGCTAACCTCCCTAT | TTATGAACTCCCAGCAACC | 237 |
| *GhDSD1.2A* | TTTTCGCTAACCTCCCTAT | ATGAACTCCCAACAACCAG | 235 |
| *GhSLD1A* | GGGTTATCGTCTAAAAGATTACG | AACACCATAGCCGAACAGA | 210 |
| *GhSLD1D* | GCAATACCTTAACAAGTTCTTCACC | CGGCTACCGATGTTAATGTA | 158 |
| *GhSLD2A* | CCATTCGTTCAGGAGCTG | CCATGAGTTTTTACTGCTTCCA | 170 |
| *GhSLD2D* | AAAGTTTCCCCATTCGTTC | AATTGGATTGCTGAGAATCCTC | 141 |
| *GhSLD3A* | GGATACAAAGTGGTTGGATAGGA | CTGTTACAAGCAATATGGTGAG | 163 |
| *GhSLD3D* | GGCGGATTCAAAGAGGTA | TATGAAAGCATCGGTGGC | 178 |
| *GhSLD4A* | GTTCACTGGTTATCATCTCAAAG | CCAAAGCAAGCCCAACAA | 229 |
| *GhSLD4D* | GTTCACTGGGTATCATCTCAAAA | ATCCAAAGCAAGCCCAAC | 231 |
| *GhSLD5* | TGGGAATTGTTTAACCGGC | GCAAGAGGGTCAAAATTCAA | 189 |
| *GhSLD5D* | TGCCATTGTTCTTTACGGTA | CCACCAGGCAATGCTGATA | 205 |
| *GhSLD5A* | TTGCCATTGTTCTTTACGGTG | CACCAGGCAATGCTGATG | 205 |
| *UBQ7* | GAAGGCATTCCACCTGACCAAC | CTTGACCTTCTTCTTCTTGTGCTTG | 198 |

**
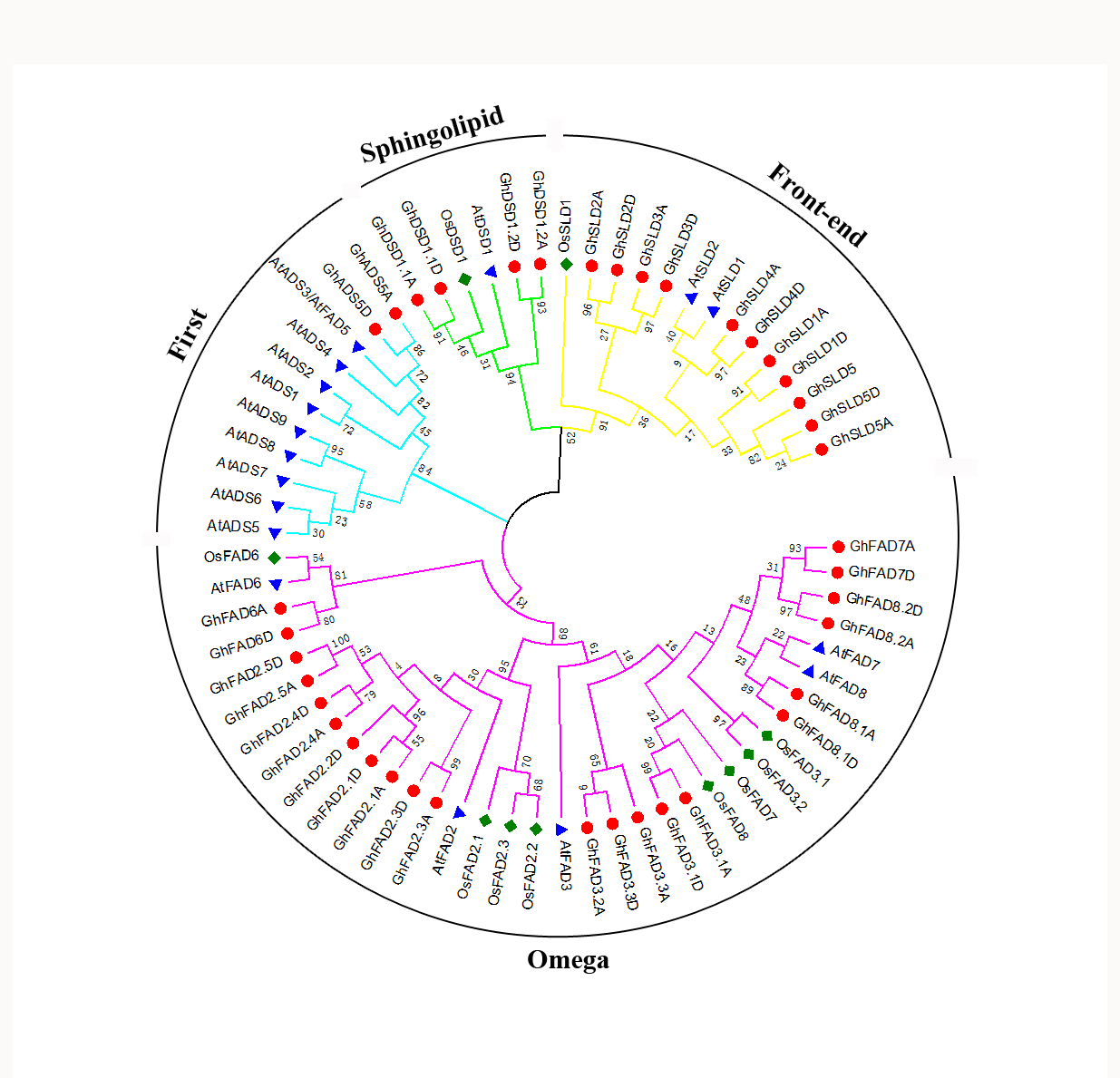
**

**Supplementary Figure S1. Phylogenetic relationships of FAD genes from *G. hirsutum*, *Arabidopsis*, and rice.** A Maximum Evolution method was used to reconstruct a phylogenetic tree of all detected FAD genes with MEGA 5.2. The Omega, First, Sphingolipid, and Front-end subfamily were marked in pink, blue, green, and yellow, respectively.

**
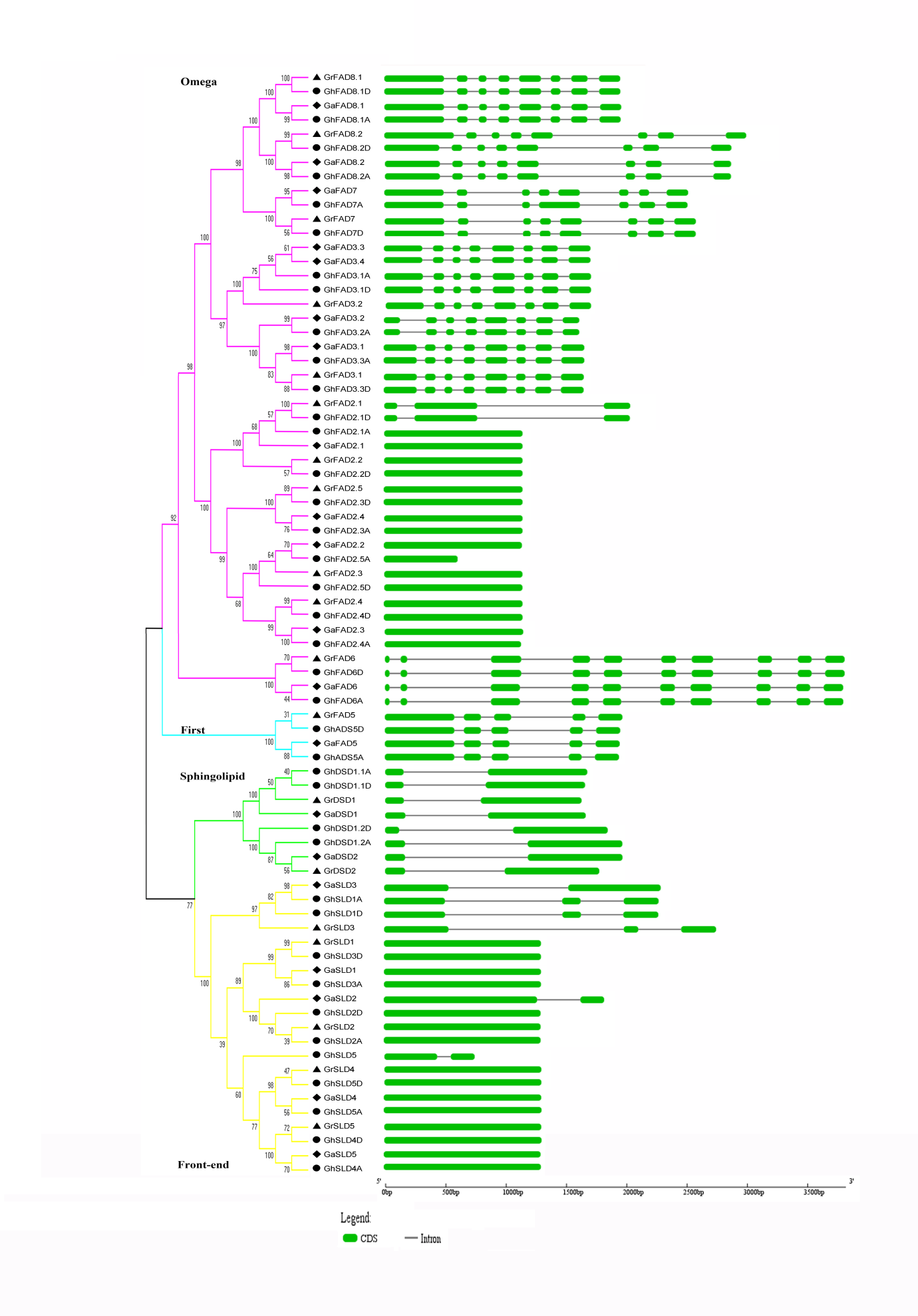
**

**Supplementary Figure S2. The structures of FAD genes from *G. hirsutum*, *G. raimondii*, and *G. arboretum*.** Exons were represented by green boxes and introns by grey lines.


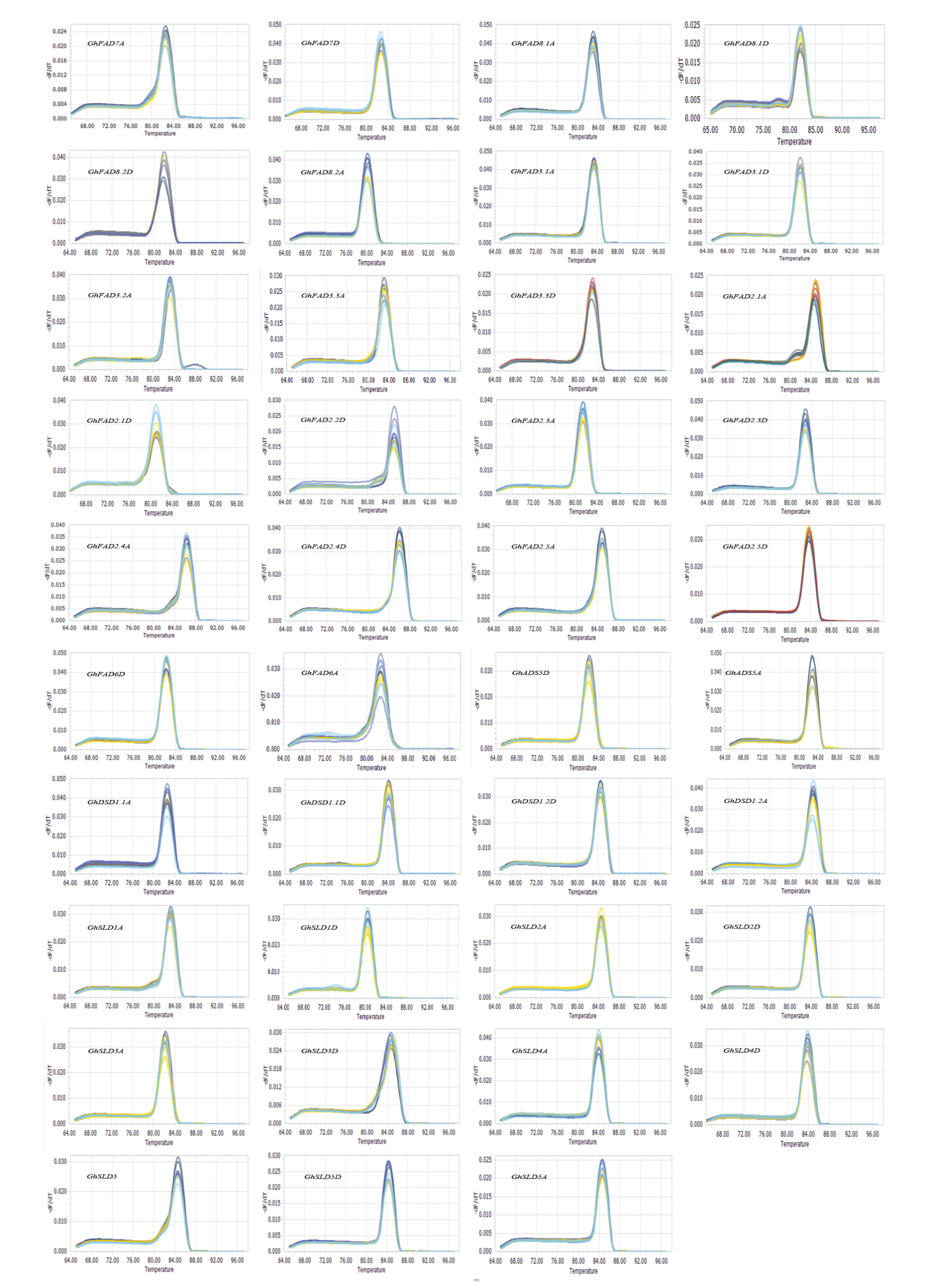


**Supplementary Figure S3. The melting curves produced by qRT-PCR.**
